# A New gcrR-Deficient *Streptococcus mutans* Mutant for Replacement Therapy of Dental Caries

**DOI:** 10.1155/2013/460202

**Published:** 2013-12-18

**Authors:** Wenting Pan, Tiantian Mao, Qing-an Xu, Jin Shao, Chang Liu, Mingwen Fan

**Affiliations:** ^1^The State Key Laboratory Breeding Base of Basic Science of Stomatology (Hubei-Most) & Key Laboratory for Oral Biomedical Engineering of Ministry of Education, School & Hospital of Stomatology, Wuhan University, 237 Luoyu Road, Wuhan 430079, China; ^2^Department of Stomatology, Renmin Hospital of Wuhan University, Wuhan University, Wuhan, Hubei 430060, China

## Abstract

*Background*. *gcrR* gene acts as a negative regulator related to sucrose-dependent adherence in *S. mutans*. It is constructive to test the potential capacity of mutans with *gcrR* gene deficient in bacteria replacement therapy. *Methods*. In this study, we constructed the mutant by homologous recombination. The morphological characteristics of biofilms were analyzed by confocal laser scanning microscopy. *S. mutans* UA159 and the mutant MS-gcrR-def were inoculated, respectively, or together for competitive testing in vitro and in rat model. *Results*. Adhesion assay showed that the adhesion ability of the mutant increased relative to the wild type, especially in the early stage. MS-gcrR-def out-competed *S. mutans* UA159 in vitro biofilm, and correspondingly coinfection displayed significantly fewer caries in vivo. The former possessed both a lower level of acid production and a stronger colonization potential than *S. mutans* UA159. *Conclusion*. These findings demonstrate that MS-gcrR-def appears to be a good candidate for replacement therapy.

## 1. Introduction

Dental caries is a well known major oral health problem in most countries. According to a national survey of the oral health status in China in 2005, more than 50% of children aged 7 years were affected by dental caries [1]. Although fluoride and other preventive methods have resulted in the decrease in dental caries, little effort has been devoted to addressing this actual pathogen that causes the infection because the oral cavity is a complex ecosystem, in which a rich and diverse microbiota has evolved. Methods such as whole bacteria replacement therapy are continuously developed to eliminate the pathogenic members of the oral cavity.

The concept of bacteria replacement therapy against dental caries has been put up early in the middle of last century [2], related to none or less virulent strains of tooth-cloning bacteria preempting the tooth surface and replacing the more cariogenic counterparts. Many relatively nonpathogenic oral commensals found in high numbers at oral cavity have been suggested as a replacement or interference organism, such as *Streptococcus sanguis* [3], *Streptococcus gordonii* [4, 5], and *Streptococcus salivarius* [6]. Unfortunately, those succedaneums cannot compete with *Streptococcus mutans* (*S. mutans*, MS) and failed in replacement therapy. The attempts to search an appropriate strain to prevent caries became focused on gene-modulation for the main pathogen, MS.

*S. mutans* is recognized as the main pathogen of dental caries in humans. In the procession development of caries, the paramount main virulence factor involves the ability to produce acid and adhere to the tooth surface [7]. In particular, the sucrose-dependent adherence of *S. mutans* to the tooth surface prevents the bacteria from being washed away by chewing or by the flow of saliva. The rationale of the sucrose-dependent adherence can be used by the low acid-producing strains to enhance their adherence ability. Thus, these strains occupy the same ecological niche in plaque similar to their more cariogenic progenitor [8]. Idone generated a mutant with gene *gcrR* knock out, a gene regulated with sucrose-dependent adherence of *S. mutans, *called GMS900 [9]. The study showed an enhancement in gene expression related to sucrose-dependent adherence in vivo, as well as significantly fewer cavities on it, which suggest the capacity of replacement therapy.

In the present study, we constructed gcrR-deficient *S. mutans* mutant and confirmed its ability of replacement therapy both in vitro and in vivo. This study demonstrated that the mutant strain, which lacks the whole open reading frame (ORF) of the *gcrR* gene, had lower acid production and stronger colonization potential compared with the wild-type strain.

## 2. Material and Methods

### 2.1. Bacterial Strains and Growth Conditions

*Escherichia coli* was grown overnight at 37°C in Luria broth (Sangon, China) with gentle aeration or on Luria agar plates supplemented with 100 *μ*g/mL of ampicillin and/or kanamycin when appropriate (Sigma-Aldrich, USA).

*S. mutans* UA159 was grown routinely as an overnight standing culture in brain-heart infusion (BHI) broth (Becton-Dickinson, USA) at 37°C in 5% CO_2_. The mutants were selected on Todd-Hewitt (TH) (Sigma-Aldrich, USA) agar plates after the strains were incubated overnight at 37°C in 5% CO_2_. Kanamycin (500 *μ*g/mL) was added to the growth medium to maintain the selective pressure for the MS-gcrR-def mutant.

### 2.2. Construction of *S. mutans* gcrR-Knockout Mutant

The details of construction of gcrR-deficient mutant are in accordance with GMS900 generated by Idone [9]. Briefly, the resulting amplicon is a 2220 bp fragment, which includes kanamycin-resistance (KanR) cassette (from pEGFP-N1 vector, 795 bp), *gcrR* upstream and downstream (720 and 705 bp from *S. mutans* UA159) at the end of KanR, and was then purified using a PCR purification kit (Qiagen, The Netherlands). The amplicon was then ligated to the pMD18-T simple plasmid to generate pMD-GK plasmid according to the manufacturer's instructions (New England Biolabs, USA).

*S. mutans* UA159 cells were transformed with the final pMD-GK plasmid in the presence of competence-stimulating peptide according to a previously described method [10]. Transformed colonies were selected on the TH-agar plate with 500 *μ*g/mL of kanamycin after these colonies were incubated overnight at 37°C in 5% CO_2_.

### 2.3. Biofilm Formation and Adherence Assay in a Monospecies Biofilm Model

*S. mutans* wild-type and mutant strains were monitored to determine their adherence to the walls of 24-well cell culture plates following 2, 4, and 6 h growths in BHI supplemented with 2% sucrose. In brief, overnight *bacteria *cultures were first standardized to 1.0 optical density (OD_470_) by using fresh physiological saline. Then the bacterial suspensions were diluted at a ratio of 1 : 20 with fresh BHI that contains 2% sucrose and distributed in triplicate in sterile 24-well cell culture plates (NEST, China) with 2.0 mL of fresh medium. The plate was kept at 37°C in 5% CO_2_ for 2, 4, and 6 h (in triplicate). Bacterial growth was determined at 470 nm by using a microplate reader. Crystal violet staining was used to monitor biofilm formation as described previously [11].

### 2.4. Confocal Laser Scanning Microscopy (CLSM) Analysis

Wild-type *S. mutans* and MS-gcrR-def mutant standardized to 1.0 OD unit were diluted at a ratio of 1 : 20 in fresh BHI with 2% sucrose. The cells were then grown on glass coverslips placed in a sterile 6-well cell culture plate (NEST, China) in 5% CO_2_ at 37°C for 2, 4, 6, and 12 h. The biofilm samples were gently rinsed thrice with PBS to remove unattached cells, dried for 3 min, and stained with molecular probes SYTO 9 (a green fluorescent cell membrane stain; Invitrogen) and propidium iodide (a red fluorescent nucleic acid stain; Invitrogen). The stained samples were then examined via CLSM (ZEISS LSM 510 META; Carl Zeiss, Germany). Images were obtained from serial optical sections and captured at 488 nm. The Z section was used to record the biofilm thickness. The red and green fluorescence intensities were quantified using Image Pro-plus 6.0 (Media Cybernetics, USA).

### 2.5. The Ability of Acid Production

After the strains were standardized to 1.0 OD unit by using physiological saline, *S. mutans* wild-type and mutant strains were diluted at a ratio of 1 : 20 in fresh BHI supplemented with 1% sucrose in triplicate. For in vitro acid production, the initial pH of the culture was measured at a constant pH of 7.3 by using a pH meter after the cell suspensions of *S. mutans*, MS-gcrR-def were mixed. The decrease in pH levels for 16 h was measured again by using a pH meter to obtain the ΔpH of these cultures.

### 2.6. Competitive Test of Biofilm Formation In Vitro

Overnight *S. mutans* UA159 cultures and the MS-gcrR-def mutant were inoculated in BHI with water (control treatment) or kanamycin and then standardized to 1.0 OD unit (OD_470_) by using fresh physiological saline. The two bacterial suspensions were diluted at a ratio of 1 : 20 with fresh BHI and distributed in 4.0 mL aliquots into sterile 6-well cell culture plates to obtain two-bacterial biofilm cells at 37°C for 4, 6, 8, 11, and 24 h. Each well was gently rinsed with distilled water to remove loose bacteria. Firmly attached cells were scraped from the substrate by pipetting and then rinsed with distilled water to ensure that all of the cells were collected. Total DNA was extracted from the same numbers of cells in the control group and the test groups by using a DNA extract kit (Qiagen, The Netherlands). Total DNA was amplified in a TaKaRa SYBR Premix system. Amplification was performed using a 7500 real-time PCR system (Applied Biosystems, USA) according to the following thermal cycling protocol: initial denaturation at 95°C for 30 s and then followed by 40 cycles of the three steps (95°C for 5 s, 51°C for 30 s, and 72°C for 60 s). Standard curves were generated for each primer-template set with a series of standard *S. mutans* UA159 genomic DNAs (ATCC, USA) that were diluted 10-fold. The standard curves were then used to calculate the correlation coefficients and translate the concentration threshold (Ct) values into the relative DNA copy number. Ct is defined as the cycle in which SYBR green fluorescence is detected at a level above the background.

### 2.7. Competitive Test of Biofilm Formation In Vivo

Eighty specific pathogen-free (SPF) female SD rats (Medical Laboratory Animal Center, Wuhan, Hubei, China) at 18 days of age were chosen for the in vivo study. In order to facilitate the cariogenic bacterial planting, all the rats were fed with appropriate antibiotics (ampicillin, chloramphenicol, and carbenicillin 1.0 g/kg diet) for three consecutive days. these groups of six rats each were challenged with oral swabs saturated with 2 × 10^9^ CFU/mL of *S. mutans* UA159, MS-gcrR-def or mixture of those two strains in ratio of 1 : 1 per mL. Over the following 3 days, the rats were challenged repeatedly with fresh swabs of the appropriate strain, and on the next day after challenge, swab samples were collected and plated onto MSB agar plated to confirm colonization. All the groups were provided caries-promoting diet [12]. Forty days after challenge, the rats were sacrificed, and their maxillary and mandibles were removed and subsequently processed for caries scores.

### 2.8. Statistical Analysis

Experimental data were analyzed on SPSS 16.0 software (SPSS, USA) and presented as means ± standard deviation. Two-group comparisons were performed using Student's *t*-test. *P* value < 0.05 was considered statistically significant.

## 3. Results

### 3.1. Confirmation of the *gcrR* Deletion in MS-gcrR-def

**Figure 1:**
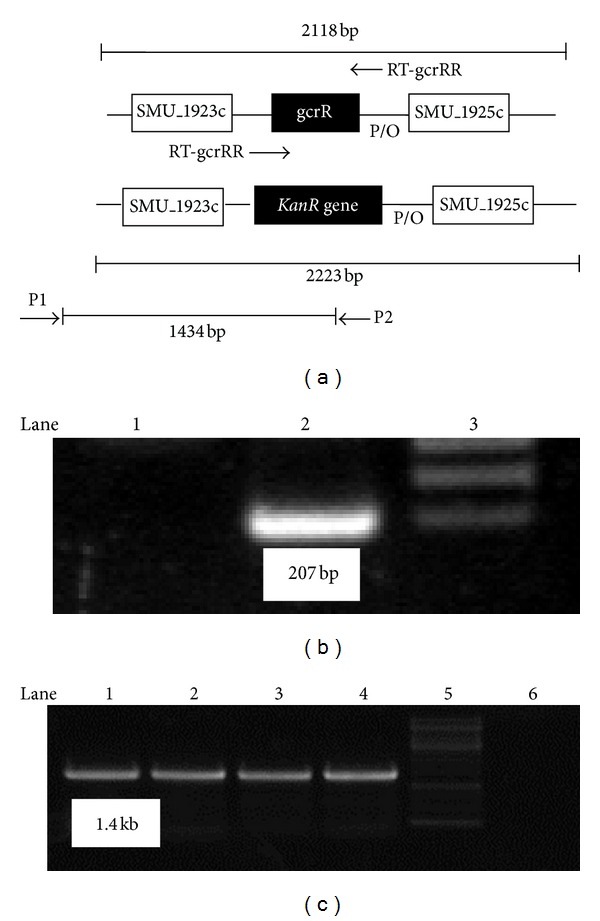
(a) To confirm that *gcrR* was deleted, chromosomal DNA was isolated from the selected transformants as well as UA159 progenitor and used as a template for PCR amplification with the P1/P2 and RT-gcrRF/RT-gcrRR primers. The resulting amplicons (the *gcrR* upstream-kanamycin resistance (KanR) cassette junction and the part of *gcrR* gene) were analyzed via agarose gel electrophoresis. Nucleotide sequencing of the purified amplicons with the same primer set was conducted to confirm the *gcrR* knockout and KanR cassette insertion. (b) Lane 1: the resulting amplicons (chromosomal DNA of MS-gcrR-def as template and RT-gcrRF/RT-gcrRR as primers); lane 2: the resulting amplicons (chromosomal DNA of UA159 as template and RT-gcrRF/RT-gcrRR as primers); lane 3: 1 kb marker (Fermentas). (c) Lanes 1, 2, 3, and 4: the resulting amplicons (chromosomal DNA of MS-gcrR-def as template and P1/P2 as primers); Lane 5, 1 kb marker (Fermentas); Lane 6, the resulting amplicons (chromosomal DNA of UA159 as template and P1/P2 as primers).

KanR transformants were screened to determine the deletion of the whole *gcrR* gene and the replacement with the KanR cassette as a result of double-crossover recombination. Chromosomal DNA was isolated from the selected transformants and UA159 progenitor, which was used as a template for PCR amplification with the P1/P2 and RT-gcrRF/RT-gcrRR primers (Table 1). The resulting amplicons (the gcrR upstream-KanR cassette junction and the part of the *gcrR* gene) were analyzed via agarose gel electrophoresis and their sizes were compared with those predicted after a successful allelic exchange event occurred (Figure 1). Nucleotide sequencing of the purified amplicons with the same primers confirmed the *gcrR* knockout and the KanR cassette insertion. The resulting mutant, which contains the *gcrR* upstream-KanR cassette junction and lacks the *gcrR* gene, was designated as MS-gcrR-def.

### 3.2. Evaluation of Biofilm Formation in Monospecies Biofilm Model

**Figure 2:**
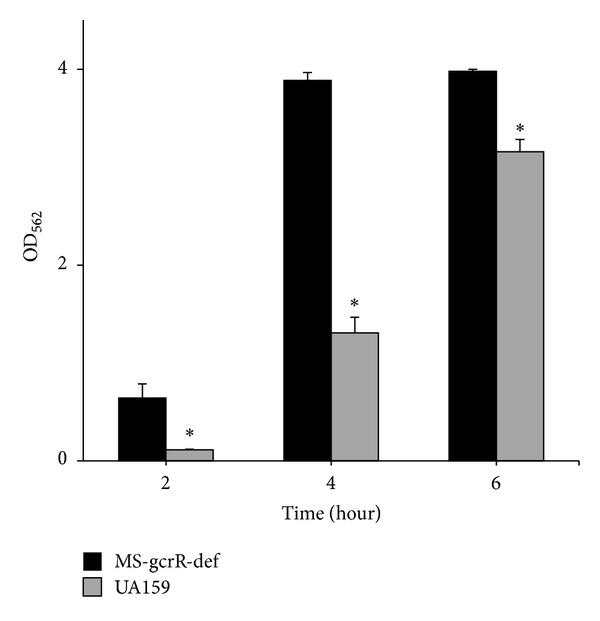
The adhesion ability of *Streptococcus mutans* UA159 as well as the MS-gcrR-def was determined based on the absorbance of the cells on the plate. *Significant differences of adhesion between UA159 and MS-gcrR-def (*P* < 0.05) were found, while there was no variance in cell growth.

We test cell growth, adherence ability, and morphology of the strains to evaluate biofilm formation. While the adhesion ability of the gcrR-deficient mutants was stronger than that of the wild type (Figure 2). Furthermore, the difference in the early stage was greater than that in the late stage. Approximately 6- and 3-fold increases at 2 and 4 h were observed in the MS-gcrR-def *gcrR* knockout mutant compared with the wild type and the compensated mutant.

**Figure 3:**
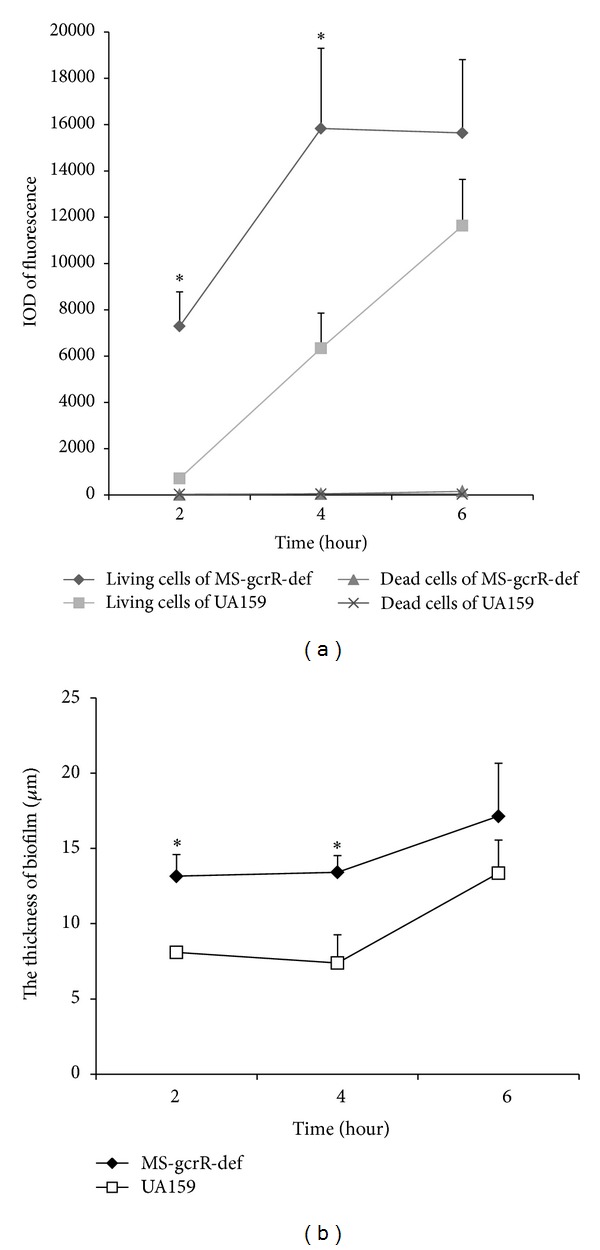
(a) IOD (integrated optical density) of the green and red fluorescence from biofilm cells. (b) Biofilm thickness. The Z section was used to record the biofilm thickness. Vertical lines were chosen randomly to analyze each image. The red and green fluorescence intensities of the dead cells and living cells, respectively, were also quantified using Image Pro-plus. *Significant difference was found between green fluorescence of UA159 and MS-gcrR-def (*P* < 0.05).

Biofilm structures were analyzed by CLSM. The top view and vertical section of the biofilm by *S. mutans* and *gcrR* deficient mutants are depicted. SYTO9 is a green fluorescent living cells membrane stain. Propidium iodide, in contrast, which is a red fluorescent nucleic acid stain penetrates only the cells with damaged membranes, thus visualizing only the dead microbes. We quantify the red and green fluorescence intensity, respectively (Figure 3). 2 h and 4 h mutant groups had conspicuous effect on increasing the number of living cells and the thickness of biofilm relative to UA159 and had statistical difference (*P* < 0.05). Accordingly, at the early stage (at 2 h and 4 h) MS-gcrR-def formed biofilms more quickly than UA159 and the biofilms were thicker relative to the wild type, which demonstrated MS-gcrR-def is a potential stronger adhesive to preempting tooth surface.

### 3.3. MS-gcrR-def Altered Acid Production Compared to UA159 Wild Type

**Figure 4:**
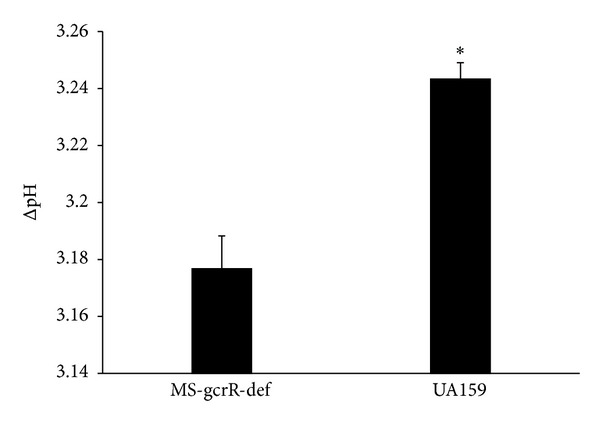
For in vitro acid production, the initial pH of the culture was measured at 7.3 by using a pH meter. Each sample was repeated three times. *Significant differences were found between UA159 and MS-gcrR-def (*P* < 0.05).

To explore potential mechanisms of hypocariogenicity of MS-gcrR-def in competition with UA159, we examined the capacity of acid production of both strains, as well as the dominate gene *ldh* in metabolism (Figure 4). The initial pH of the culture when mixed with *S. mutans* and MS-gcrR-def was 7.3 and the final pH of the above two cultures was significantly lower than those values for the culture of MS-gcrR-def which were in the range of 4.11–4.13 after 16 h of incubation at 37°C. The MS-gcrR-def had a small average ΔpH which was substantially smaller than the average ΔpH of the wild type (*P* < 0.05).

### 3.4. MS-gcrR-def Was Competitive Than UA159 In Vitro

**Figure 5:**
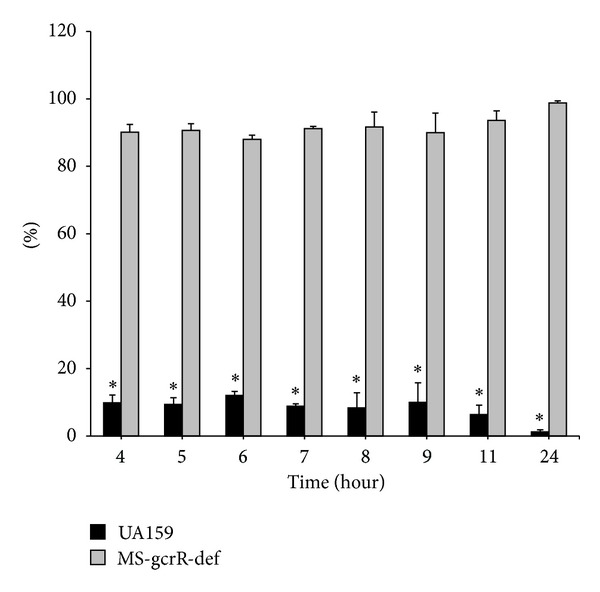
The coculture bacteria were harvested and used to extract RNA; expression of biofilm-associated genes was examined by quantifying the *gcrR* gene using real-time RT-PCR. *Significant difference observed from comparison of the two groups at every testing time (*P* < 0.05).

To certify the dominance in dual-species biofilm in replacement therapy, we inoculated the two strains into polystyrene plates with identical initial condition. The mutant exhibited an advantage over the wild type when these strains were cultured together. In particular, the gcrR-defective bacteria accounted for >90% in all of the adherent bacteria (Figure 5). Thus, the initial advantage continued to increase over time.

### 3.5. MS-gcrR-def Was Competitive Than UA159 in Germfree Rats

**Figure 6:**
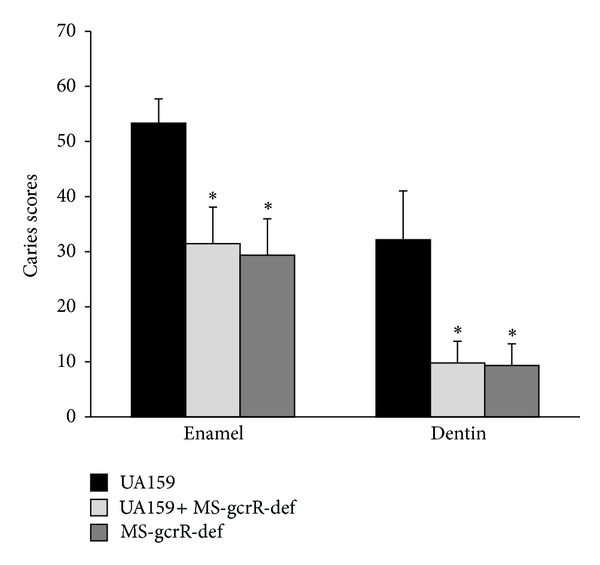
The enamel and dentinal caries scores of rats infected with UA159, MS-gcrR-def, and the mixture of both. The caries levels were scored by Keyes' method. The results are expressed as mean ± S.D. values. Symbols for statistical significance: *significantly different from the group UA159 infection alone (*P* < 0.05).

Since there is conspicuous superiority of MS-gcrR-def in the dual-bacteria biofilm, the mixing bacteria model in germfree rats is constructed to confirm its hypocariogenic ability in vivo. The rats are sacrificed 40 days after initial inoculation. The depth of carious lesion in both maxillary and mandibles involvement was scored for statistics (Figure 6). The duel-bacteria group and MS-gcrR-def monospecies group displayed significantly fewer enamel and dentinal lesions than UA159 wild-type group (*P* < 0.05). There was no significant difference in dental caries lesions between dual-bacteria group and MS-gcrR-def monospecies group (*P* > 0.05).

## 4. Discussion 

In the present study, we addressed the relationship both in vitro and in vivo of two strains, UA159 and MS-gcrR-def, and indicated a potential effector strain in bacterial replacement therapy. It was introduced to us with the increasing desire for natural products for health maintenance. For many years, replacement therapy has been considered a promising approach for the prevention of certain microbial infections [13]. Parenterally administered competitive strains indiscriminately boycott pathogenic bacteria species associated with the host disease, resulting in formation of ecological volume exclusion and resistance development. Besides, the strains with the capacity of excluding colonization and/or infection could be considered as controlled microflora-composition modulation that otherwise occurs haphazardly in nature [14].

*S. mutans* is by far the most common pathogen found in dental caries and may get spontaneous mutation in environmental induction. Directly implant the harmless bacteria which are strongly competitive with *S. mutans*, leading to achieving tailor-made protection for the host against it. Since the effector strain is closely contacted to the host, herd protection through natural transmission might be increased too. Jeffrey Hillman's group generate a hypocariogenic strain which affects the acid production of *S. mutans* by using recombinant DNA methodology and persistently continue the research for 20 years [15]. Mutant only exhibiting defects in acid production and hypocariogenicity in animal models are unlikely to compete successfully with wild-type strains for initial plaque locations [16]. In this case, we contemplate to make an appropriate gene modification in *S. mutans* to develop the effector strain with ecological volume exclusion and low acid production.

Glucan-binding lectin (GBL), which enables bacteria to aggregate by binding the *α*-1,6-glycosidic linkages, is ubiquitous on the *S. mutans* surface. GBL is an adhesin and can be isolated from many *S. mutans *subtypes [17]. With the help of adhesions such as GBL, *S. mutans* can synthesize many kinds of glucan to form a wrap of mucosal fluid, which promotes its tight adherence to the tooth surface. Although different opinions on the mechanism by which GBL functions have been presented, GBL has a very important function in the formation of dental plaque and adherence of *S. mutans *[18]. The *gcrR* gene functions as a negative transcriptional regulator of the *gbpC* gene, which encodes the *S. mutans* GBL. Some researchers revealed that the *gcrR* expression product shares a high homology with the signaling molecule of the density inductance system, which may be involved in the regulation of the GBL expression. For instance, Sato et al. [10] revealed the downregulation of *gbpC* gene by *gcrR* gene. On the contrary, the *gcrR* gene knockout results in the overexpression of GBL. In the study of Idone et al., RT-PCR demonstrated an increase in gtfD, *gbpC* gene of strain GM900, leading to augment in sucrose-dependent adherence. Our group did the same research on MS-gcrR-def and got the identical result (data not shown). Interestingly, they suggested the aggregate of GM900 increase in vitro, while it reveals compromise in their ability to colonize in vitro model of tooth surface and to rat molars than the wild type. We have the converse result to the point. In our research, comparing with UA159, MS-gcrR-def had evident superiority in sucrose-dependent adherence to borosilicate glass and absolutely dominated in the biofilm construction, indicating strong competitiveness of the former by the weight of evidence. It may be due to the strain of *S. mutans* and culture method.

In this case, the deficiency of gene *gcrR* results in consecutive descent in pH value of MS-gcrR-def cells, as well as the biofilm it forms. In 2008, Dunning and colleagues showed that GMS901, the *S. mutans* mutant, which harbors insertion-deletion mutation in *gcrR*, is acid sensitive and has a significantly lower acid tolerance response (ATR) compared with the wild-type progenitor strain *S. mutans* UA159 [19]. They suggested *gcrR* could increase the expression of gene *ffh* and gene *atpA/E* which related to ATR and may modulate the pH value through proton-translocating ATPase on the cell membrane [20]. In the acidic environment, it is crucial to maintain transmembrane pH values so that the intracellular pH compared to the environment is alkaline, which is benefit for the glycolytic activity of the cariogenic bacteria [21].

Taken together, MS-gcrR-def showed reduced acid production and significantly enhanced adhesion ability at the early stage, which might confer it an absolute advantage in the competition with wild type. These findings suggest that MS-gcrR-def is a promising effect strain in place of UA159. In the replacement therapy, even if the wild-type strain interference cannot be avoided, the mutant, with its strong early colonization ability, is still able to become the dominant bacteria on the surface of tooth and reduce the incidence of dental caries.

## Figures and Tables

**Table 1:** Primers used in identification of the combination.

Primer name	Primer sequences (5′–3′)	Tm (°C)	Applications	Amplicon size
P1	AAAGCCCAGCAGCATCAC	56	Verifying the correct mutant	1434 bp
P2	TGCCGAGAAAGTATCCATCA			
RT-gcrRF	CAGCTCGGTTATGAACATCT	52	RT-PCR of *gcrR *	207 bp
RT-gcrRR	TTATCACTGCTCGTGACTCTAT			
